# Durable clinical and metabolic response to Trastuzumab Deruxtecan in heavily Pretreated male HER2-low metastatic breast cancer: a case report

**DOI:** 10.1093/omcr/omaf182

**Published:** 2025-12-26

**Authors:** Oumaima Lamsyah, Intissar Belrhali, Yousra Al Harrak, Majdouline Bel Lakhdar, Sihame Lkhoyaali, Saber Boutayeb, Ibrahim El Ghissassi, Hind M’rabti, Hassan Errihani

**Affiliations:** Department of Medical Oncology, National Institute of Oncology (INO), Ibn Sina University Hospital, Faculty of Medicine and Pharmacy, Mohammed V University, Avenue Allal El Fassi,Souissi, 10100 Rabat, Rabat-Salé-Kénitra Region, Morocco; Department of Medical Oncology, National Institute of Oncology (INO), Ibn Sina University Hospital, Faculty of Medicine and Pharmacy, Mohammed V University, Avenue Allal El Fassi,Souissi, 10100 Rabat, Rabat-Salé-Kénitra Region, Morocco; Department of Medical Oncology, National Institute of Oncology (INO), Ibn Sina University Hospital, Faculty of Medicine and Pharmacy, Mohammed V University, Avenue Allal El Fassi,Souissi, 10100 Rabat, Rabat-Salé-Kénitra Region, Morocco; Department of Nuclear Medicine, Ibn Sina University Hospital, Faculty of Medicine and Pharmacy, Mohammed V University, Street Mfadel Cherkaoui, Souissi, 10100 Rabat, Rabat-Salé-Kénitra Region, Morocco; Department of Medical Oncology, National Institute of Oncology (INO), Ibn Sina University Hospital, Faculty of Medicine and Pharmacy, Mohammed V University, Avenue Allal El Fassi,Souissi, 10100 Rabat, Rabat-Salé-Kénitra Region, Morocco; Department of Medical Oncology, National Institute of Oncology (INO), Ibn Sina University Hospital, Faculty of Medicine and Pharmacy, Mohammed V University, Avenue Allal El Fassi,Souissi, 10100 Rabat, Rabat-Salé-Kénitra Region, Morocco; Department of Medical Oncology, National Institute of Oncology (INO), Ibn Sina University Hospital, Faculty of Medicine and Pharmacy, Mohammed V University, Avenue Allal El Fassi,Souissi, 10100 Rabat, Rabat-Salé-Kénitra Region, Morocco; Department of Medical Oncology, National Institute of Oncology (INO), Ibn Sina University Hospital, Faculty of Medicine and Pharmacy, Mohammed V University, Avenue Allal El Fassi,Souissi, 10100 Rabat, Rabat-Salé-Kénitra Region, Morocco; Department of Medical Oncology, National Institute of Oncology (INO), Ibn Sina University Hospital, Faculty of Medicine and Pharmacy, Mohammed V University, Avenue Allal El Fassi,Souissi, 10100 Rabat, Rabat-Salé-Kénitra Region, Morocco

**Keywords:** trastuzumab deruxtecan, male breast cancer, HER2-low, gender disparity, antibody-drug conjugate

## Abstract

**Background:**

Male breast cancer (MBC), accounting for < 1% of all breast malignancies, remains underrepresented in clinical trials evaluating human epidermal growth factor receptor 2 (HER2)-targeted therapies. Most MBCs are hormone receptor-positive (HR+), with a high rate of HER2-low expression. However, men were excluded from clinical trials evaluating HER2-directed agents, which is an absolute evidence gap.

**Case Report:**

We present the case of a 76-year-old man with heavily pretreated, HR+/HER2-low (immunohistochemistry [IHC] 1+) metastatic breast cancer (mBC) who achieved a complete metabolic response (CMR) following four cycles of trastuzumab deruxtecan (T-DXd) without any grade ≥ 2 (G ≥ 2) toxicity.

**Conclusion:**

This case highlights the potential efficacy and tolerability of T-DXd in men and challenges the systematic exclusion of male patients from clinical trials involving HER2-low mBC. We advocate routine HER2-low testing and gender-inclusive trial design.

## Introduction

Male breast cancer (MBC) is rare, representing 0.6–1% of all breast cancers, with a 40% increase in incidence between 1975 and 2015 [1, 2]. Despite obvious biological distinctions such as high hormone receptor (HR) positivity and low HER2 expression, treatment strategies are primarily guided by data from female patients, as men are systematically underrepresented in clinical trials. Regulatory agencies have acknowledged this disparity and now support male inclusion in breast cancer trials [3].

HER2-low breast cancers, defined as IHC 1+ or 2+ with negative in situ hybridization (ISH), represent approximately 50% of HER2-negative cases and have demonstrated sensitivity to HER2-targeted antibody-drug conjugates (ADCs) [2]. Trastuzumab deruxtecan (T-DXd) significantly improved progression-free survival (PFS) in HER2-low breast cancer in the DESTINY-Breast04 trial (10.1 vs. 5.4 months; HR = 0.51), although men made up less than 1% of the trial cohort [4–6].

We report a case of a 76-year-old man with HER2-low metastatic MBC with a durable response following eight cycles of T-DXd, supporting the need for broader male recruitment in future trials.

## Case presentation

### Initial diagnosis (2008)

A 76-year-old man with no significant comorbidities, was diagnosed with left invasive breast carcinoma in 2008 (22 × 12 mm, grade II, HR+, HER2 3+) and treated with mastectomy, adjuvant chemotherapy, radiotherapy, and tamoxifen (discontinued due to thromboembolism).

### First relapse (2020)

In 2020, he presented with progressive dyspnea, fatigue, and weight loss. Thoraco-abdominopelvic computed tomography (CT) revealed bilateral subcentimetric pulmonary nodules. Positron emission tomography (PET-CT) revealed hypermetabolic foci suggesting metastasis. Metastatic adenocarcinoma consistent with primary breast cancer was diagnosed through atypical lingula resection (HR+, HER2 IHC 1+, ISH-negative according to 2018 ASCO/CAP guidelines).The treatment history prior to initiation of trastuzumab deruxtecan is summarized in Table 1.

**Table 1:** Treatment history prior to Trastuzumab Deruxtecan.

Therapy Line	Agent	Best Response	Reason for Discontinuation	PFS (months)
First-line	Paclitaxel	Complete response (PET)	Disease progression	6
Second-line	Eribulin	Stable disease	Radiographic progression	3
Third-line	Capecitabine	Partial response	Biochemical progression (CA 15–3 ↑53%)	13
Fourth-line	Vinorelbine	Stable disease	Disease progression	5
Fifth-line	Gemcitabine	Stable disease	Grade 3 fluid retention	4

**CA15–3:** Cancer Antigen 15–3; **PFS:** Progression-free survival; **PET:** Positron emission tomography

**Grade 3:** Severe symptoms requiring medical intervention, in accordance with CTCAE v5.0.

### Initiation of trastuzumab deruxtecan

In April 2024, T-DXd was initiated at 5.4 mg/kg every 3 weeks owing to symptomatic nodal progression and rising CA 15–3 levels to 42.8 IU/ml.

**Figure 1:**
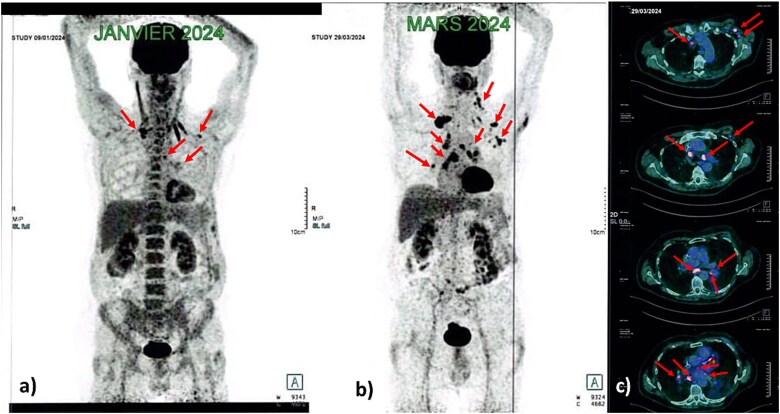
MIP view of FDG PET/CT from January 2024 (**a**) and march 2024 (**b**) showing metabolic progression of supradiaphragmatic nodal spread (arrows). Axial views of FDG PET/CT from march 2024 (**c**) showing hypermetabolic foci in the mediastinal and left axillary lymph nodes. **MIP:** Maximum intensity projection, **FDG:** Fluorodeoxyglucose, **PET/CT:** Positron emission tomography/computed tomography.

**Figure 2:**
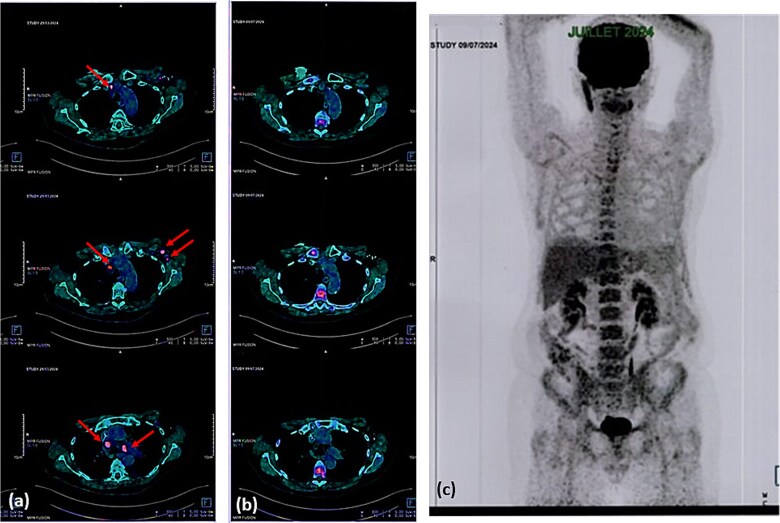
Comparative FDG PET/CT study between march 2024 (**a**) and July 2024 (**b**) showing complete metabolic regression of the hypermetabolic foci in the mediastinal and left axillary lymph nodes (arrows). MIP view from July 2024 (**c**). **FDG:** Fluorodeoxyglucose, **PET/CT:** Positron emission tomography/computed tomography, **MIP:** Maximum intensity projection.

**Figure 3:**
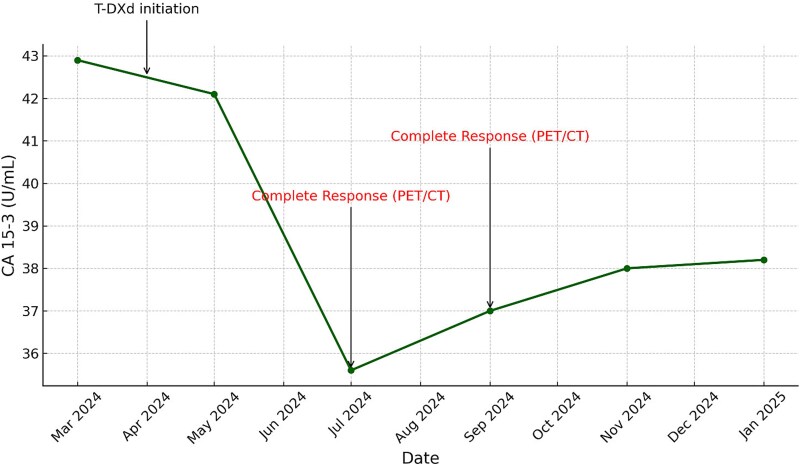
Longitudinal CA 15–3 levels during trastuzumab deruxtecan therapy. **CA 15–3:** Cancer antigen 15–3; **PET/CT:** Positron emission tomography/computed tomography. **Complete response** was defined by PET/CT metabolic assessment (FDG uptake disappearance of hypermetabolic lesions).

By cycle 4, PET/CT showed a complete metabolic response and CA 15–3 declined to 35.6 U/ml. By cycle 8, a complete metabolic response was maintained (Figs 1 and 2). At cycle 13, isolated right supraclavicular hyperfixation (SUVmax 2.8) was observed without clinical correlation or biochemical progression (CA 15–3: 38.2 U/ml). Given the sustained clinical benefit, absence of interstitial lung disease (ILD), and only grade 1 toxicities reported in cycle 14, a multidisciplinary team elected to continue T-DXd. A definitive reassessment of the disease status is pending further PET/CT evaluation. The longitudinal response metrics and toxicity profiles are summarized in Table 2 and Fig. 3.

**Table 2:** Treatment response and adverse events under trastuzumab deruxtecan.

Timepoint	CA 15–3 (U/mL)	Radiologic/Clinical Response	Adverse Events	Therapeutic Decision
Baseline	42.8	Multiple hypermetabolic foci	None	T-DXd initiation
Cycle 4	35.6	Complete metabolic response (PET)	Grade 1 nausea, Grade 1 fatigue	Continue
Cycle 8	37.0	Complete metabolic response (PET)	Grade 1 alopecia	Continue
Cycle 13	38.2	Stable disease, isolated SUV 2.8	None	Continue

**CA 15–3:** Cancer Antigen 15–3; **PET/CT:** Positron Emission Tomography/Computed Tomography; **SUV:** Standardized Uptake Value; **T-DXd:** trastuzumab deruxtecan.

**Grade 1:** Mild, asymptomatic or mild symptoms not requiring intervention, in accordance with CTCAE v5.0.

## Discussion

This case provides rare clinical evidence of a durable response to T-DXd in male HER2-low MBC, a population excluded from pivotal trials. The complete metabolic response after five failed therapies, with sustained PFS exceeding 10 months, emphasizes the potential of HER2-low targeting in men.

According to current guidelines and pivotal clinical trials, trastuzumab deruxtecan is recommended as a second-line or later-line therapy for patients with HER2-low metastatic breast cancer [4].

Male breast cancers exhibit distinct biological features, including nearly universal HR positivity and a high prevalence of HER2-low expression. In a recent prospective multicenter study by Ignatov et al. (2024) analyzing 870 MBC cases, HER2-low tumors were identified in 76% of men, compared to 55% of women [7]. Similarly, Shang et al. (2025) reported HER2-low or ultra-low expression in 67.9% of 106 male breast tumors, significantly higher than rates observed in female cohorts (*P* < 0.05). Although HER2 status did not correlate with overall survival, the predominance of HR-positive, HER2-low tumors in older men may create a tumor microenvironment particularly responsive to ADCs [8].

Preclinical evidence suggests that estrogen signaling enhances HER2 membrane recycling, thereby amplifying the bystander effect of T-DXd, especially in hormone-rich tumors [9]. This could explain the prolonged PFS observed in our patient, exceeding the median of 10.1 months reported in DESTINY-Breast04, despite extensive prior treatment [4].

The DESTINY-Breast06 trial further supports the utility of T-DXd in HER2-low and HER2-ultralow tumors, showing a median PFS of 13.2 months versus 8.1 months with chemotherapy. Although overall survival data remain immature, these findings suggest that HER2-low targeting may extend to broader biological subsets [10].

The observed safety profile further underscores potential sex-specific mechanisms. Despite prior exposure to myelosuppressive agents and pulmonary metastases, the patient experienced no ILD and only mild (grade 1) toxicities. This contrasts with the DESTINY-Breast04 trial, which 12.1% developed ILD and 52% had grade ≥ 3 adverse events [4].

The isolated supraclavicular PET-positive lesion in cycle 13 highlights a key diagnostic challenge in ADC-treated patients, distinguishing true progression from immune or inflammatory phenomena. In line with evolving ADC treatment paradigms, clinical-radiological concordance has guided the decision to continue therapy. This case highlights the value of integrating patient-reported outcomes and biochemical markers when interpreting equivocal imaging findings.

This case raises important considerations that challenge current assumptions in breast oncology:

- HER2-low biology may not be sex-specific. The pattern of HER2 expression in men warrants further investigation.
- The exclusion of male patients from clinical trials is no longer justified. This case provides practical evidence to support the inclusion of men in HER2-low breast cancer studies.
- ILD is not necessarily inevitable with T-DXd. Our observations suggest that sex-related pharmacokinetic factors or biological differences may contribute to a more favorable toxicity profile.

Overall, this case highlights that a HER2-low status represents a clinically actionable target in MBC. This finding also supports the need for routine assessment of HER2-low expression in male patients. We advocate for a minimum representation of 5% male patients in breast cancer clinical trials to help reduce longstanding gender disparities in oncologic research.
